# Design and implementation of a practical quality control program for dual‐energy CT

**DOI:** 10.1002/acm2.13396

**Published:** 2021-09-02

**Authors:** Crystal A. Green, Justin B. Solomon, Kenneth J. Ruchala, Ehsan Samei

**Affiliations:** ^1^ Department of Radiology Clinical Imaging Physics Group Duke University Medical Center Durham North Carolina USA; ^2^ Clinical Imaging Physics Group Carl E. Ravin Advanced Imaging Laboratories Duke University Medical Center Durham North Carolina USA; ^3^ Sun Nuclear Corporation Middleton Wisconsin USA; ^4^ Department of Radiology Clinical Imaging Physics Group Carl E. Ravin Advanced Imaging Laboratories Duke University Medical Center Durham North Carolina USA

**Keywords:** calcium quantification, dual‐energy CT, iodine quantification, quality control, spectral CT

## Abstract

A novel routine dual‐energy computed tomography (DECT) quality control (QC) program was developed to address the current deficiency of routine QC for this technology. The dual‐energy quality control (DEQC) program features (1) a practical phantom with clinically relevant materials and concentrations, (2) a clinically relevant acquisition, reconstruction, and postprocessing protocol, and (3) a fully automated analysis software to extract quantitative data for database storage and trend analysis. The phantom, designed for easy set up for standalone or adjacent imaging next to the ACR phantom, was made in collaboration with an industry partner and informed by clinical needs to have four iodine inserts (0.5, 1, 2, and 5 mg/ml) and one calcium insert (100 mg/ml) equally spaced in a cylindrical water‐equivalent background. The imaging protocol was based on a clinical DECT abdominal protocol capable of producing material specific concentration maps, virtual unenhanced images, and virtual monochromatic images. The QC automated analysis software uses open‐source technologies which integrates well with our current automated CT QC database. The QC program was tested on a GE 750 HD scanner and two Siemens SOMATOM FLASH scanners over a 3‐month period. The automated algorithm correctly identified the appropriate region of interest (ROI) locations and stores measured values in a database for monitoring and trend analysis. Slight variations in protocol settings were noted based on manufacturer. Overall, the project proved to provide a convenient and dependable clinical tool for routine oversight of DE CT imaging within the clinic.

## 1 INTRODUCTION

The current standard for routine quality control for computed tomography (CT) is defined according to the American College of Radiology's (ACR) CT QC Manual. This manual describes a quality control (QC) program with daily, monthly, and annual testing components to better ensure consistent quality and output of a CT imaging system. Likewise, the American Association of Physicists in Medicine (AAPM) Task‐Group 233 offers a comprehensive evaluation of CT performance.^2^ However, neither of these resources offer evaluation methods for dual‐energy CT (DECT) systems. DECT systems utilize a second energy source or detector to acquire projection data, with dedicated hardware and postprocessing analyses deployed for the detection, visualization, and quantification of various elements, tissues, and features.^3^, ^4^, ^5^ These unique capabilities cannot be monitored under the current standards of quality control for conventional CT. Thus, there is a need for a routine dual‐energy quality control (DEQC) program to ensure the accuracy and reproducibility of DECT data and associated applications.

One of the disadvantages of conventional CT is that materials with different elemental composition can be represented by the same CT number. This poses great difficulty in classifying and distinguishing different tissue types. Using material decomposition, attenuation measurements from the second energy source allow a mixed material to be decomposed into two or three base materials. This allows the creation of synthetic image types such as iodine quantification maps, virtual monoenergetic images, virtual noncontrast (iodine‐subtracted) images. The blended (high kV/low kV) images allow prominent features both from the high kV (e.g., reduction in noise and improved image sharpness) and low kV (e.g., low attenuating objects) to be seen in the same view. Material quantification (i.e., iodine or calcium quantification) can be indicative of lesion enhancement or hemorrhaging depending on the anatomical application. Material removal techniques are helpful for a radiologist to better visualize unenhanced areas for iodine subtraction or better visualize vasculature with calcium removal of plaques for CT angiography.^6^

A routine DEQC program will require a phantom, specific scan protocol, and data analysis archival for routine quantification and monitoring over time. In terms of phantoms, currently there exists several multi‐energy CT phantoms. The current commercial offerings allow a wide variety of material inserts to verify quantitative accuracy and some offer larger frames (up to 40 cm for body protocols). However, these current offerings are not conducive to practical routine clinical use. In terms of protocols, Nute et al. highlighted scan parameters and test metrics for DECT characterization and intra‐system variablity.^7^ Jacobsen et al. further identified errors and differences across multiple vendors for iodine quantification and monochromatic attenuation.^8^ However, the intra‐system variability for material inserts as well as the exact methodology for assessing phantom attributes hampers, complicates, and confuses the clinical implementation.

The objective of this study was to introduce a methodology and the evaluation for the design and implementation of a DEQC program. The goal for this project was a streamlined solution that would be focused on routine QA monitoring on a weekly or bi‐weekly basis. The proposed QC program aimed at (1) the prototype of a simple and affordable phantom with clinically relevant materials and concentrations, (2) a clinically relevant acquisition, reconstruction, and postprocessing protocol, and (3) a fully automated analysis software to extract quantitative data for database storage and trend analysis over time.

## 2 METHODS

The methodology for creating a DEQC program can be divided into three parts: (1) the phantom design and development, (2) the protocol development, and (3) the automated analysis and initial routine testing.

### 2.1 The DECT phantom

Using phantoms for quality control is an established practice, and many of the traditional QA tests (e.g., image uniformity, HU accuracy) will still be applicable to DECT. Work from Nowik et al. shows the importance of a CTQC program detecting various changes in key performance indicators for CT, which include the detection of changes in CT numbers.^9^ However, a DECT phantom used for routine QC will require additional agents to ensure accurate and consistent measurements specific to DECT. Some key considerations in our project were (1) the use of a water equivalent background to ensure HU accuracy, (2) clinically relevant inserts that are iodine based at various concentrations, (3) a calcium insert to ensure identification of bone and other calcified plaques, and (4) practicality in terms of size and overall ease of use in an already established QC program.

It was imperative that the phantom design should include clinically relevant iodine concentrations (mg I/ml) that are often used as discrimination points for various diagnosis across DECT technologies. Various studies use 0.5 mg I/ml as a threshold to determine the enhancement of vascular and nonvascular lesions.^10^, ^11^, ^12^ Additionally, a phantom based intra‐manufacturer study by Jacobsen et al. found 0.5 mg I/ml (small phantom) and 1.0 mg I/ml (large phantom) as the limit of detection for the various systems tested.^13^ A study by Patel et al. found that normalizing iodine quantification can better separate vascular and nonvascular renal lesions based on variation of the type of DECT technology used (kV switching vs. dual source). That study found the mean optimal absolute discriminant to be 1.5 mg I/ml and normalized discriminant values to be 0.3 mg I/ml.^14^

Several studies used dual‐source DECT for improved detection and discrimination using iodine quantification. An iodine quantification study by Kaltenbach et al. found that using both iodine uptake (>2.9 mg I/ml) and normalized iodine uptake (>0.22) to be helpful in distinguishing between hepatic neuroendocrine tumor metastasis from hepatocellular carcinoma.^15^ Additionally, Martin et al. found the optimal threshold for diagnosis of acute pancreatitis to be 2.1 mg I/ml with a sensitivity of 96% based on a 45 patient study.^16^ Xu et al. found that in addition to other morphological features, iodine quantification and iodine ratios were key predictors in differentiating small‐cell lung cancer from nonsmall cell lung cancer. Small‐cell lung cancer had lower iodine ratios and lower iodine density concentrations (1.17 ± 0.28 mg I/ml) versus nonsmall‐cell lung cancer (1.55 ± 0.47 mg I/ml).^17^

Additionally, several studies used rapid kV switching DECT for improved detection and discrimination using iodine quantification. A study by Yamauchi et al. found significant differences between values for benign (mean 1.16 mg I/ml) and malignant (mean 2.32 mg I/ml) post‐treatment head and neck tumors using DECT iodine concentration maps and monoenergetic derived images at 40 keV and 70 keV.^18^ Additionally, a cardiac study by Hur et al. found that DECT was highly sensitive in the detection of the left atrial appendage thrombi and differentiation between thrombus (1.23 ± 0.34 mg I/ml) and spontaneous echo contrast echo pattern (3.61 ± 1.01 mg I/ml) in patients with stroke.^19^

**FIGURE 1:**
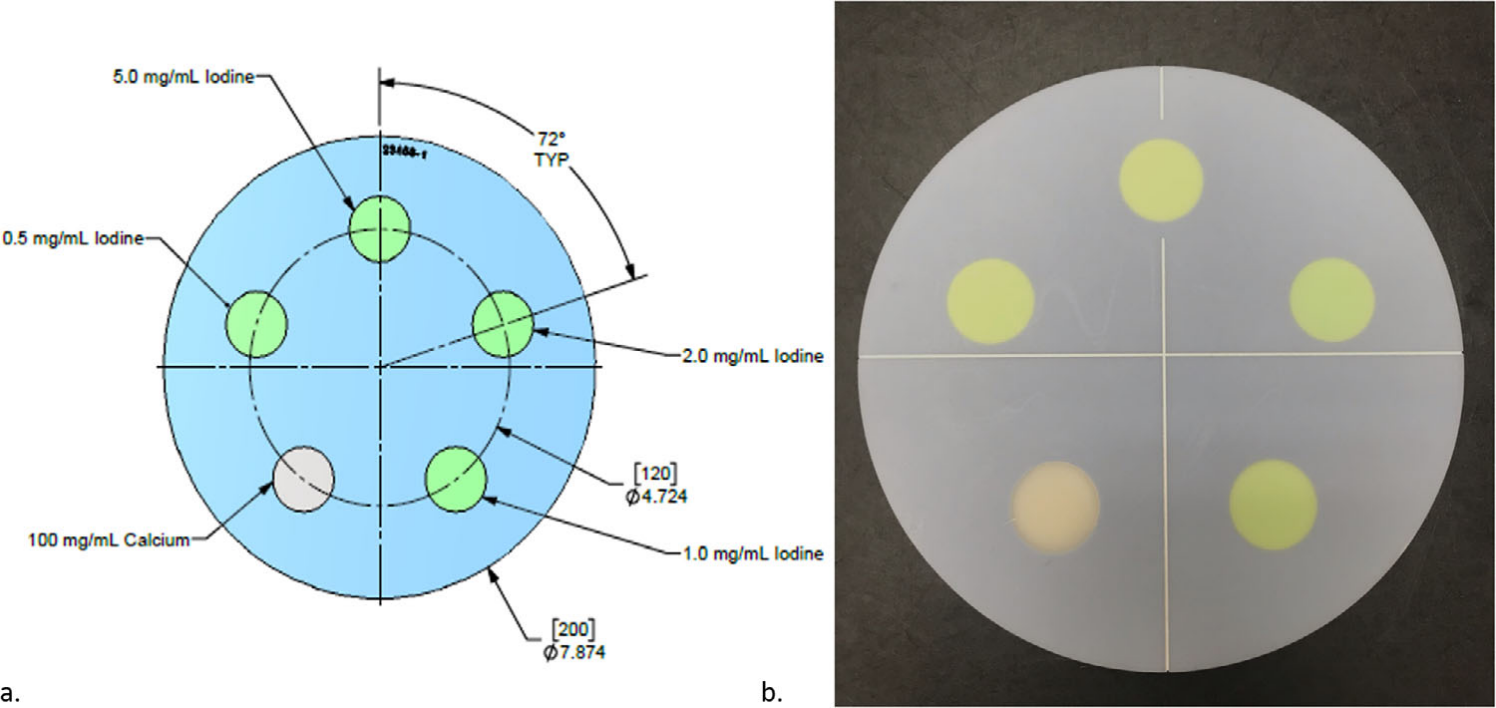
(a) Schematic of dimensions and insert locations of the dual‐energy computed tomography (DECT) phantom; (b) physical representation of the DECT phantom

**FIGURE 2:**
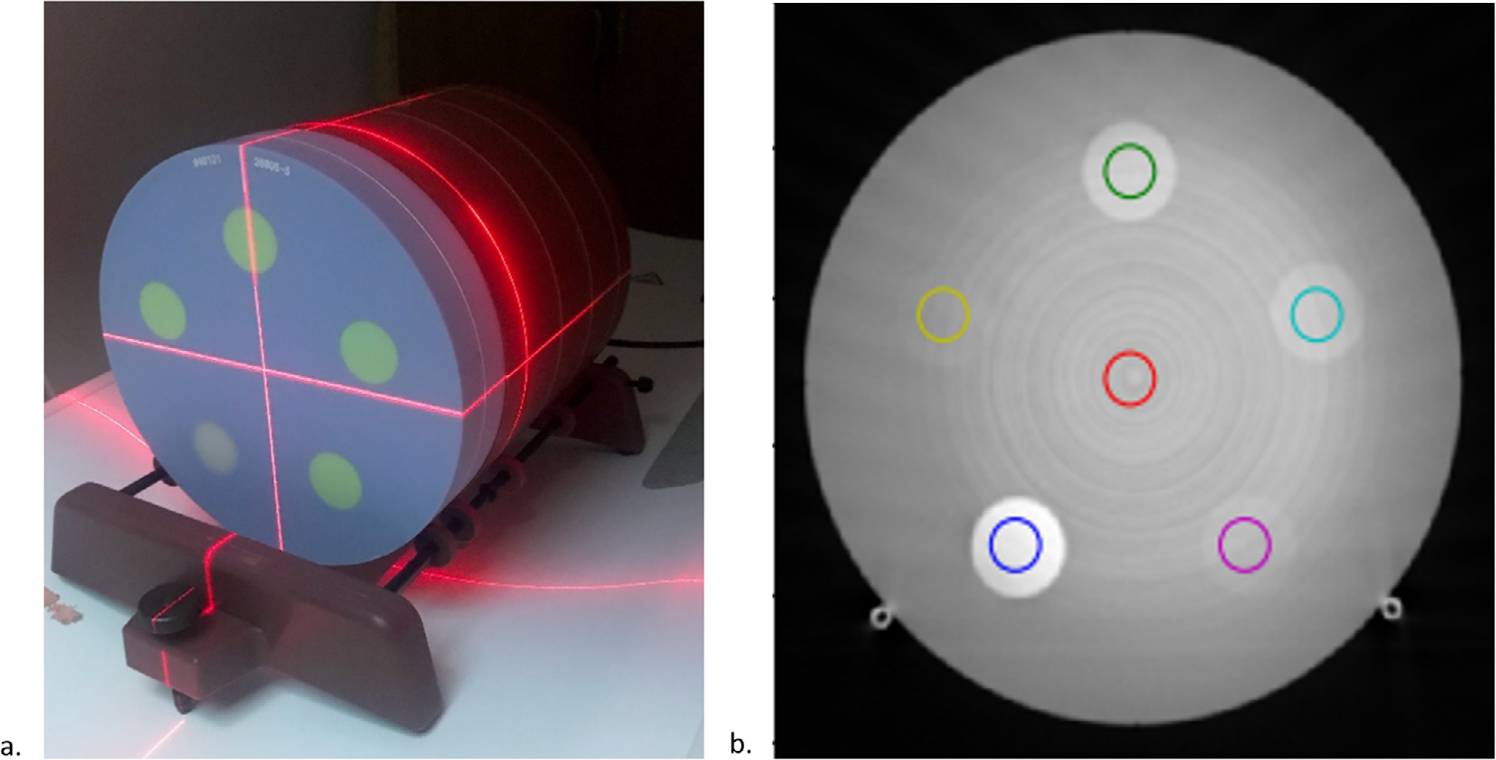
(a) Recommended phantom setup adjacent to ACR phantom; (b) axial slice of acquired dual‐energy computed tomography (DECT) phantom images scanned alone. CT Ring artifacts are present and ROI locations are shown for each insert

Based on literature review and clinical assessments, the DECT phantom prototype was designed in collaboration with Sun Nuclear Corporation (given constraints of cost and practicality) to contain four iodine inserts (0.5, 1, 2, and 5 mg I/ml) and one calcium insert (100 mg Ca/ml), each with a diameter of 2.8 cm, equally spaced in a cylindrical water‐equivalent background material. The final design is shown in Figure 1. The 100 mg Ca/ml insert, and the 5 mg I/ml were slated to be nonadjacent since those contain the two largest HU values. All inserts were fixed within the phantom to aid in cost effectiveness. The phantom's iodine concentrations were chosen based on being in the ranges of iodine enhancement (0.5 mg I/ml) and clinical discrimination values as described previously from literature review. The calcium concentration was chosen based on being the intermediate concentration offered by the phantom manufacturer. The phantom was fabricated to be 20 cm in diameter and 4 cm in depth to facilitate imaging adjacent to the ACR phantom with the use of an extended stand (as shown in Figure 2a) or standalone, which allows DECT phantom to be scanned coincidently with a site's ACR QC. The phantom uses a new blue‐colored high equivalency (HE) CT Solid Water, Model 1451 (Sun Nuclear Corporation).

For the assessment of the virtual monoenergetic images, the theoretical Hounsfield units were calculated and provided by the manufacturer. The material compositions of the phantom materials were entered into the National Institute of Standards and Technology (NIST) XAAMDI database (NIST 5632 report), where the respective mass attenuation coefficients from 40 to 150 keV were determined.^20^ Using Equation 1, the Hounsfield units, listed in Table 1, were calculated as

(1) $$\mathrm{HU}\left(E\right)=\;1000\;\frac{\rho_{X}\;.\left[\;\frac{\mu }{\rho }\left(E\right)\right]-\mu {\left(E\right)}_{\mathrm{WATER}}}{\mu {\left(E\right)}_{\mathrm{WATER}}},$$

where *ρ_X_* is the density of the phantom insert, $\frac{\mu }{\rho }\left(E\right)$ is the NIST mass attenuation coefficient of the material at a specific energy E, and $\mu {\left(E\right)}_{\mathrm{WATER}}$ is the linear attenuation coefficient of water at a specific energy E.

**TABLE 1:** NIST calculated Hounsfield units for dual‐energy computed tomography (DECT) phantom prototype inserts for 40–150 keV energy intervals, based on liquid water at standard temperature and pressure (STP)

Energy (keV)	Iodine 0.5 mg/ml (HU)	Iodine 1.0 mg/ml (HU)	Iodine 2.0 mg/ml (HU)	Iodine 5.0 mg/ml (HU)	Calcium 100 mg/ml (HU)	1451 HE CT solid water (HU)
40	44.7	86.0	170.4	422.6	675.7	−2.1
50	29.3	56.5	112.2	278.3	485.1	−3.0
60	19.0	37.4	75.2	187.6	378.1	−4.3
70	12.6	25.5	52.2	131.5	316.3	−5.1
80	9.0	18.4	38.1	96.2	278.9	−5.2
90	6.4	13.6	28.5	72.6	254.7	−5.3
100	4.7	10.3	22.0	56.4	239.0	−5.5
110	3.6	8.0	17.4	45.0	228.6	−5.4
120	2.6	6.2	13.9	36.5	220.6	−5.6
130	1.8	4.8	11.3	30.1	214.5	−5.8
140	1.2	3.7	9.2	25.2	209.8	−5.9
150	0.8	3.0	7.8	21.5	206.3	−5.8

Abbreviation: HE CT, high equivalency computed tomography.

### 2.2 Imaging protocol

Generally, manufacturers have designed default DECT protocols that could be utilized as part of a routine DEQC program. However, these protocols may not be representative of how DECT is used clinically at specific facilities. Additionally, manufacturer protocols may not be vendor‐neutral due to the differences in DECT hardware and data processing methods. Some key considerations to our site‐specific DECT protocol development were (1) iodine/calcium material quantification/removal accuracy, (2) CT accuracy of virtual monoenergetic images, (3) and CT accuracy of polychromatic (blended or mixed) images. As DECT aims to discriminate materials, the testing protocol should be made in consideration of the accuracy of material quantification and the technology deployed. The imaging protocol for this study was developed for two scanner types, fast kV switching^21^ and dual source.^22^

Irrespective of manufacturer, energy‐independent, material‐specific, and material removed synthetic images can be derived using material decomposition of acquired DECT data. Virtual monoenergetic images, also referred to as virtual mono‐chromatic images, are reconstructions that mimic the appearance of the CT images with a true monoenergetic photon source between 40 and 200 keV.^5^ Material‐specific images are often presented as a distribution map based on a materials mass density. Common materials used are iodine/water or calcium/water for concentration maps. Material removed images refer to the images where the iodine or calcium has been removed which may be referred to as virtual noncontrast (VNC) or calcium‐removed images, respectively, dependent on the manufacturer. Our protocol included these renditions of the DECT data from the phantom.

The protocol development initially started with our site's DECT abdominal protocol and was accordingly adjusted to fit our QC needs. Initially, we acquired data using both helical and axial scans at the widest beam width, 5 mm slices, 120 blended kV, and 220 mm detector field of view. Axial scans proved to be more sensitive for the identification of potential issues. Since our site acquires abdominal monoenergetic images at 70 keV, that value was used accordingly.

The protocols were developed and implemented on Siemens SOMATOM Definition Flash and GE Discovery CT 750 HD scanners. Detailed descriptions of the imaging protocol are shown in Table 2. Since the two scanners use different DECT hardware and postprocessing technologies, there were slight variations to the protocol based on scanner type. For example, calcium quantification images were not processed due to unavailability in current Siemens software processing for Siemens DECT acquisitions. Similarly, material suppressed images (iodine subtraction) are not available for the GE image acquisitions. GE's AW server can generate several types of synthetic maps (i.e., material suppressed images) and other material density combinations, which has been utilized in other works.^23^, ^24^ However, that feature is not included in the clinical work‐flow tool, and thus requires manual processing which is impractical for a routine automated QC program.

**TABLE 2:** Finalized protocol description based on scanner type

CT scanner type	Protocol description	
Siemens SOMATOM Definition Flash (dual source)	Scan description:	
	kV A/B Tube = 100/140 (Sn)	mAs A/B Tube = 320/248
	Acquisition: Axial	Slice thickness = 5 mm (128 × 0.6 mm)
	Rotation time = 0.5 s	Display FOV = 220 mm
	CTDIvol (32 cm body) = 24.64 mGy	Reconstruction Kernel: QR40
	Reconstructions:	
	A Tube Projection	B Tube Projection
	Mixed Projection (A+B at 0.5 DE compensation),	Liver VNC (Iodine‐Water Material Density)*
	Liver VNC (Virtual Unenhanced)*	Monoenergetic (70 keV)*
GE Discovery CT750 HD (rapid kV switching)	Scan Description:	
	kV: 80/140 (GS‐15)	mAs: 640
	Acquisition: Axial	Slice Thickness: 5 mm (5/8i)
	Rotation time = 0.6 s CTDIvol (32 cm body) = 21.5 mGy	Display FOV = 220 mm Reconstruction Kernel = STD
	Reconstructions:	
	Mixed Projection	Monoenergetic (70 keV) ^+^
	Iodine (Water) Material Density^+^ (iodine quantification)	Calcium (Water) Material Density Map^+^ (calcium quantification)

* 3 mm reconstructed slices.

^+^ 2.5 mm reconstructed slices.

### 2.3 The DECT automated algorithm and workflow

**FIGURE 3:**
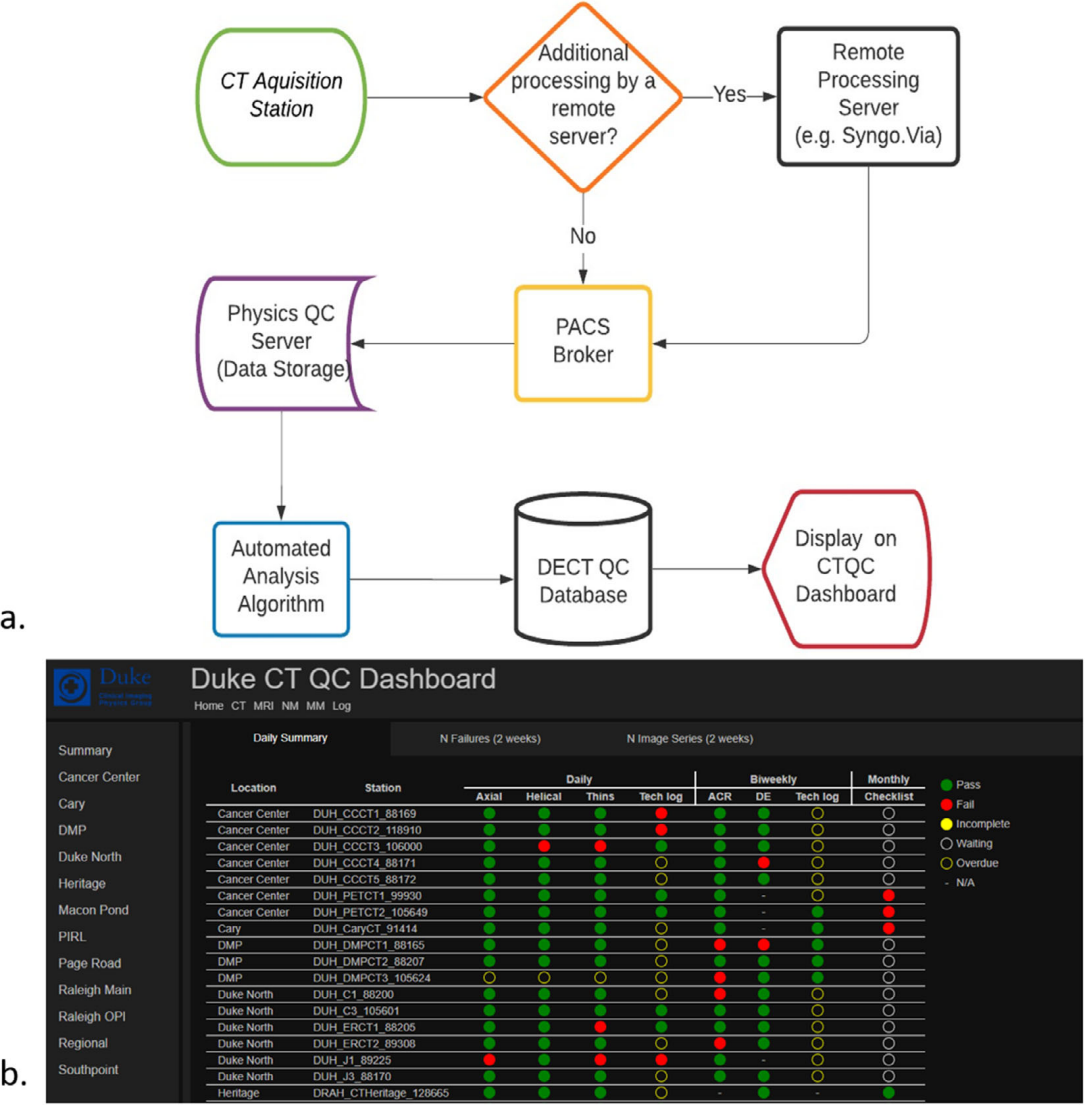
(a) Flowchart of dual‐energy computed tomography quality control (DECT QC) workflow; (b) example of CT QC dashboard

Once the phantom is scanned, a designated workflow is needed to ensure the images and reconstructions are fully processed and make it to a server for access. Our QC automated analysis software used open‐source technologies integrated into our current automated CT QC database. For Siemens datasets, all DECT reconstructions are processed on a remote server (SyngoVia). Thus, all the synthetic reconstructions must be properly routed to ensure they make it to the QC server. For GE, reconstructions are processed on the scanner. An overview of the DECT QC workflow is shown in Figure 3a.

The process was designed such that once a study arrives at the Physics QC server, an automated algorithm will process the acquired data and store the results in a database. This algorithm was written using open‐source technology (python version 3.9.0). First, the automated algorithm reads in the monoenergetic image series study and detects the relevant slice location of the DECT phantom. If for some reason the monoenergetic series is unavailable, the mixed kV series is used instead. Next, the algorithm locates the 100 mg Ca/ml insert location using a thresholding technique. Once the center of the calcium insert is located, the algorithm determines the center points of the other phantom inserts based on the fixed geometric and angular dependencies from the overall phantom design from the calcium insert. Then, the algorithm uses a 15 mm diameter ROI (shown in Figure 2b) and measures the mean, standard deviation, minimum and maximum values for each material insert over a central 25 mm slice range. This process is repeated for all relevant reconstructed images depending on manufacturer type as described in the reconstruction section of Table 2. The results for each study are stored in the DEQC database, which resides on our Physics QC server. Database results can be viewed on the CTQC dashboard where trends and alert thresholds can be reviewed and assessed.

### 2.4 QC implementation

The methodology was applied for 3 months on the two scanner models noted above. For all, the DECT phantom was scanned adjacent to the Gammex 464 ACR CT Accreditation phantom (Figure 2a). Additionally, routine DEQC can be monitored using the CTQC dashboard as shown in Figure 3b. The image data were analyzed in terms of the various reconstructed DECT image types: mixed/blended kV, monoenergetic at 70 keV, iodine quantification, iodine removal (virtual noncontrast), and calcium quantification.

## 3 RESULTS

### 3.1 Mixed/blended kV analysis

**FIGURE 4:**
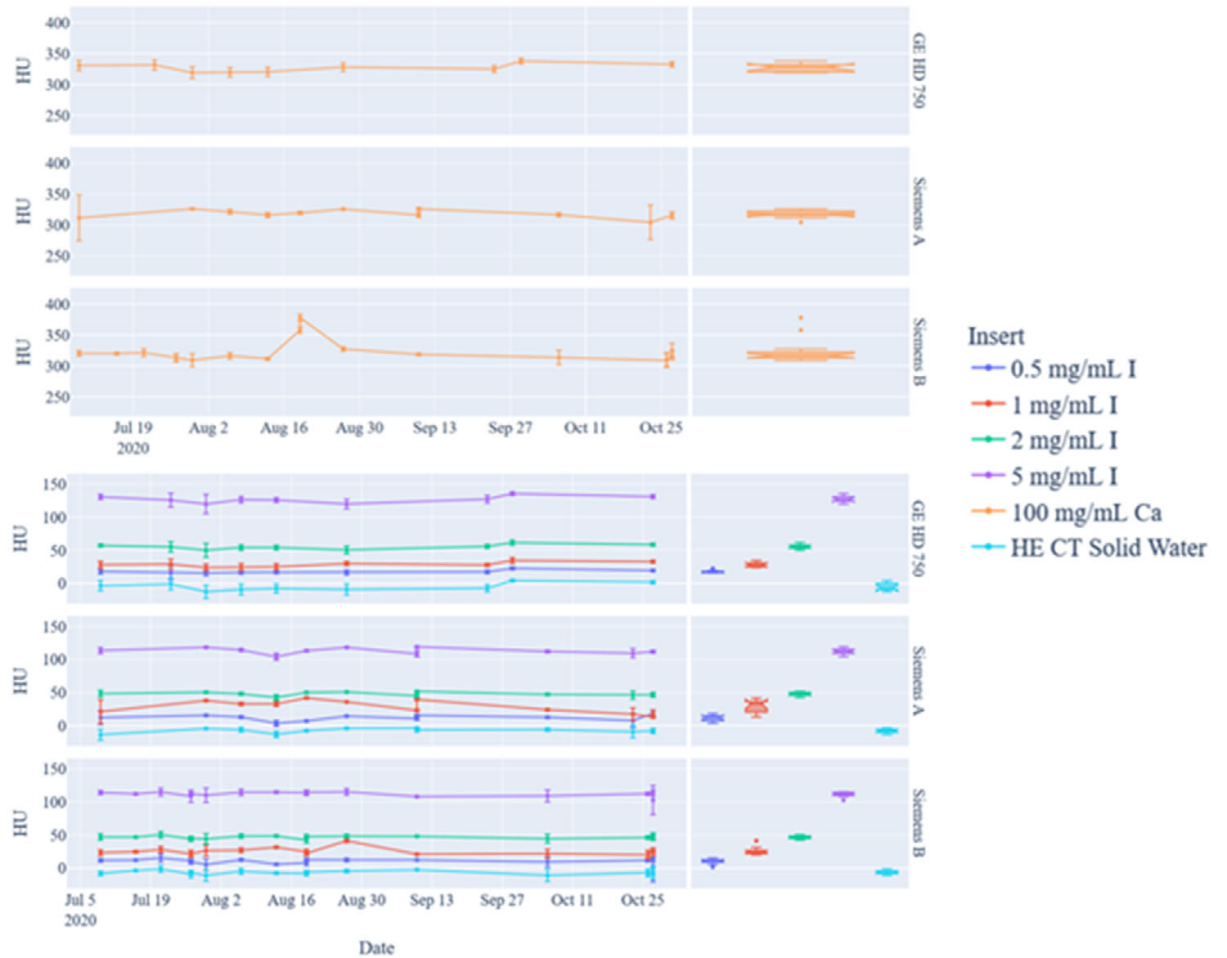
Dual‐energy quality control (DEQC) analysis of the dual‐energy computed tomography (DECT) phantom for blended dual energy images based on phantom inserts (top) calcium‐insert only (bottom) iodine inserts and solid water background (left) scatterplot showing mean HU and standard deviation values for each insert over a 3‐month period for the specified phantom inserts (right) box plot of measured values

The mixed kV (blended) DEQC results are shown in Figure 4 and Table 3. The average HU values (mean ± SD) measured across all systems over the 3‐month timeframe based on phantom insert were 12.4 ± 4.6 HU (0.5 mg I/ml), 27.0 ± 6.7 HU (1 mg I/ml), 48.9 ± 4.5 HU (2 mg I/ml), 115.2 ± 7.6 HU (5 mg I/ml), 321.9 ± 13.2 HU (100 mg Ca/ml), and −6.7 ± 3.9 HU (water equivalent material).

**TABLE 3:** Mixed kV results for all phantom inserts across the three dual‐energy computed tomography (DECT) scanners

	Mixed kV results (mean ± SD HU)					
	DECT phantom insert					
Station ID	100 mg Ca/ml	0.5 mg I/ml	1 mg I/ml	2 mg I/ml	5 mg I/ml	Solid water
GE HD 750	327.1 ± 5.6	17.7 ± 2.2	28.4 ± 3.7	55.2 ± 3.6	126.9 ± 5.1	−4.9 ± 5.6
Siemens A	317.8 ± 6.1	11.4 ± 4.6	28.5 ± 9.4	47.4 ± 2.9	112.1 ± 4.8	−7.9 ± 3.5
Siemens B	322.4 ± 18.2	10.4 ± 3.4	25.1 ± 5.1	46.6 ± 2.1	111.9 ± 3.3	−6.4 ± 2.7
Total	321.9 ± 13.2	12.4 ± 4.6	27.0 ± 6.7	48.9 ± 4.5	115.2 ± 7.6	−6.7 ± 3.9

### 3.2 Monoenergetic image analysis

**FIGURE 5:**
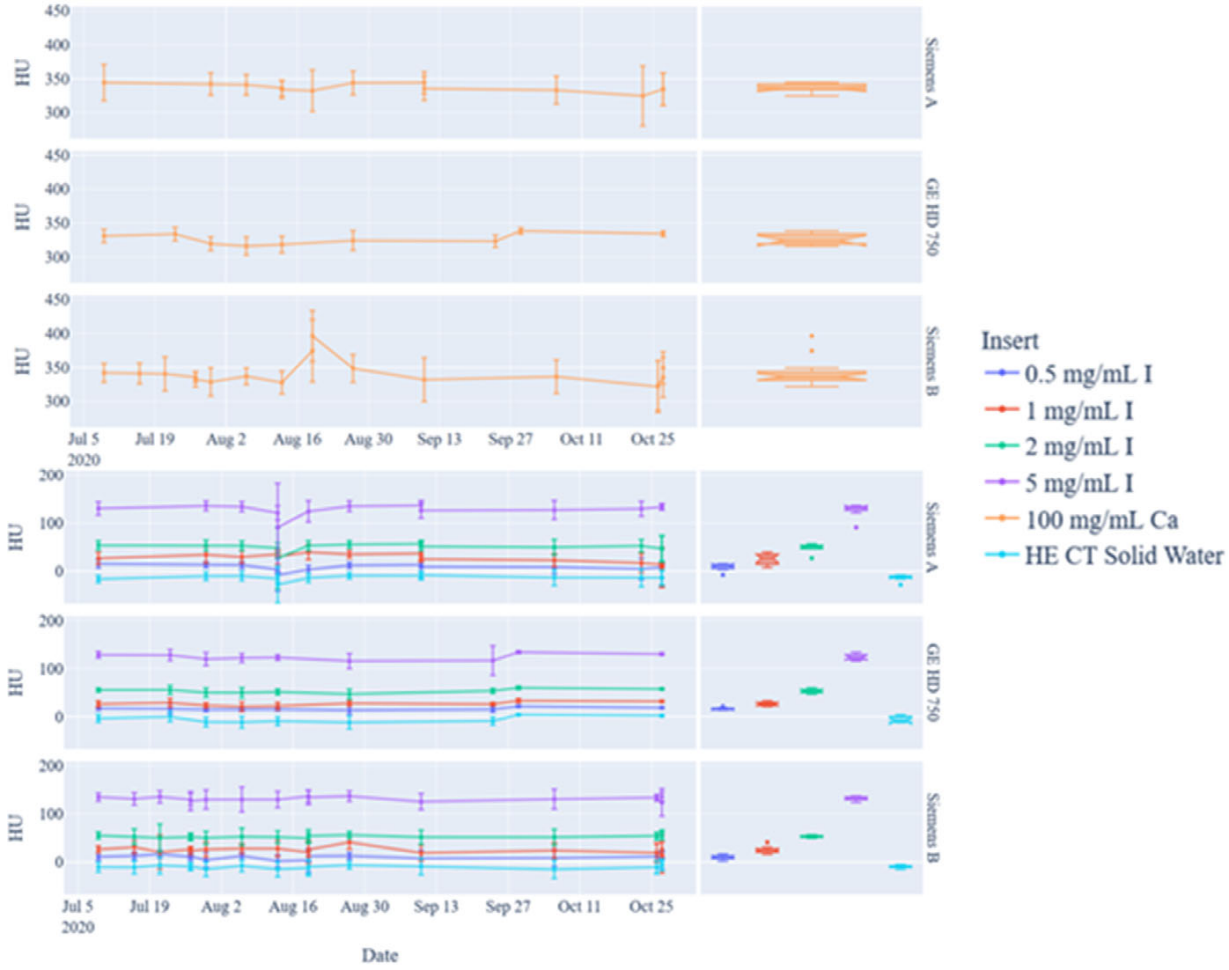
Dual‐energy quality control (DEQC) analysis of the dual‐energy computed tomography (DECT) phantom for 70 keV monoenergetic synthetic images based on phantom inserts (top) calcium‐insert only (bottom) iodine inserts and solid water background (left) scatterplot showing mean HU and standard deviation values for each insert over a 3‐month period for the specified phantom inserts (right) box plot of measured values

Figure 5 and Table 4 show the results of our DEQC for 70 keV synthetic reconstructions. The average HU values (mean ± SD) measured across all systems over the 3‐month timeframe based on phantom insert were 11.0 ± 5.4 HU (0.5 mg I/ml), 25.0 ± 7.9 HU (1 mg I/ml), 51.6 ± 5.0 HU (2 mg I/ml), 128.6 ± 8.2 HU (5 mg I/ml), 336.3 ± 14.0 HU (100 mg Ca/ml) and −10.5 ± 5.3 (water equivalent material).

**TABLE 4:** Monoenergetic (70 keV) results for all phantom inserts across the three dual‐energy computed tomography (DECT) scanners

	70 keV monoenergetic results (mean ± SD HU)					
	DECT phantom insert					
Station ID	100 mg Ca/ml	0.5 mg I/ml	1 mg I/ml	2 mg I/ml	5 mg I/ml	Solid water
GE HD 750	326.5 ± 8.0	16.2 ± 2.4	26.5 ± 4.2	53.1 ± 4.2	123.9 ± 6.3	−5.9 ± 6.3
Siemens A	336.5 ± 5.5	9.13 ± 6.19	24.5 ± 11.3	49.3 ± 7.0	128.2 ± 11.2	−13.6 ± 4.7
Siemens B	341.2 ± 18.7	9.8 ± 4.2	24.6 ± 5.8	52.7 ± 2.1	131.5 ± 3.9	−10.2 ± 2.9
Total	336.3 ± 14.0	11.0 ± 5.4	25.0 ± 7.9	51.6 ± 5.0	128.6 ± 8.2	−10.5 ± 5.3

### 3.3 Material quantification and removal analysis

**FIGURE 6:**
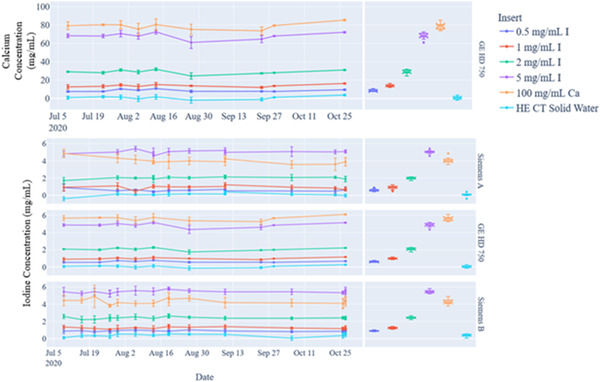
Dual‐energy quality control (DEQC) analysis of the dual‐energy computed tomography (DECT) phantom for calcium quantification (top) and iodine quantification (bottom 3) (left) scatterplot showing mean mg/ml and standard deviation values for each insert over a 3‐month period (right) box plot of measured values

Figure 6 and Table 5 show the results of our DEQC for iodine and calcium material quantification. The average iodine density (mg I/ml) values (mean ± SD) measured across all systems over the 3‐month timeframe based on phantom insert were 0.7 ± 0.2 mg I/ml (0.5 mg I/ml), 1.1 ± 0.2 mg I/ml (1 mg I/ml), 2.2 ± 0.2 mg I/ml (2 mg I/ml), 5.2 ± 0.3 mg I/ml (5 mg I/ml), 4.5 ± 0.7 mg I/ml (100 mg Ca/ml), and 0.2 ± 0.2 mg I/ml (water equivalent material). Calcium quantification could only be performed on the GE system. The average calcium density (mg Ca/ml) values (mean ± SD) measured across all systems over the 3‐month timeframe for the 100 mg Ca/ml insert was 78.6 ± 3.5 mg Ca/ml.

**TABLE 5:** Iodine quantification, calcium quantification, and virtual noncontrast (iodine removal) results across applicable scanners

	Iodine map results (mean ± SD mg I/ml)					
	DECT phantom insert					
Station ID	100 mg Ca/ml	0.5 mg I/ml	1 mg I/ml	2 mg I/ml	5 mg I/ml	Solid water
GE HD 750	5.6 ± 0.3	0.6 ± 0.1	1.0 ± 0.1	2.1 ± 0.2	4.9 ± 0.3	0.1 ± 0.1
Siemens A	4.0 ± 0.3	0.6 ± 0.1	0.9 ± 0.2	2.0 ± 0.1	5.0 ± 0.2	0.1 ± 0.2
Siemens B	4.3 ± 0.3	0.9 ± 0.1	1.2 ± 0.1	2.4 ± 0.1	5.4 ± 0.2	0.4 ± 0.1
Total	4.5 ± 0.7	0.7 ± 0.2	1.1 ± 0.2	2.2 ± 0.2	5.2 ± 0.3	0.2 ± 0.2

	Calcium map results (mean ± SD mg Ca/ml)					
	DECT phantom insert					
Station ID	100 mg Ca/ml	0.5 mg I/ml	1 mg I/ml	2 mg I/ml	5 mg I/ml	Solid water
GE HD 750	78.8 ± 3.6	8.6 ± 1.3	13.8 ± 1.4	28.8 ± 2.3	68.1 ± 3.6	0.78 ± 1.7

	Virtual noncontrast (mean ± SD HU)					
	DECT phantom insert					
Station ID	100 mg Ca/ml	0.5 mg I/ml	1 mg I/ml	2 mg I/ml	5 mg I/ml	Solid water
Siemens A	227.31 ± 5.6	−6.0 ± 5.3	4.2 ± 8.4	−1.2 ± 6.8	−1.5 ± 8.4	−14.2 ± 5.3
Siemens B	214.5 ± 46.0	−12.3 ± 4.9	−7.0 ± 5.7	−9.1 ± 4.2	−8.2 ± 5.0	−18.4 ± 3.5
Total	220.1 ± 36.2	−9.6 ± 6.1	−2.3 ± 9.0	−6.1 ± 6.2	−5.7 ± 7.3	−17.1 ± 4.4

Abbreviation: DECT, dual‐energy computed tomography.

**FIGURE 7:**
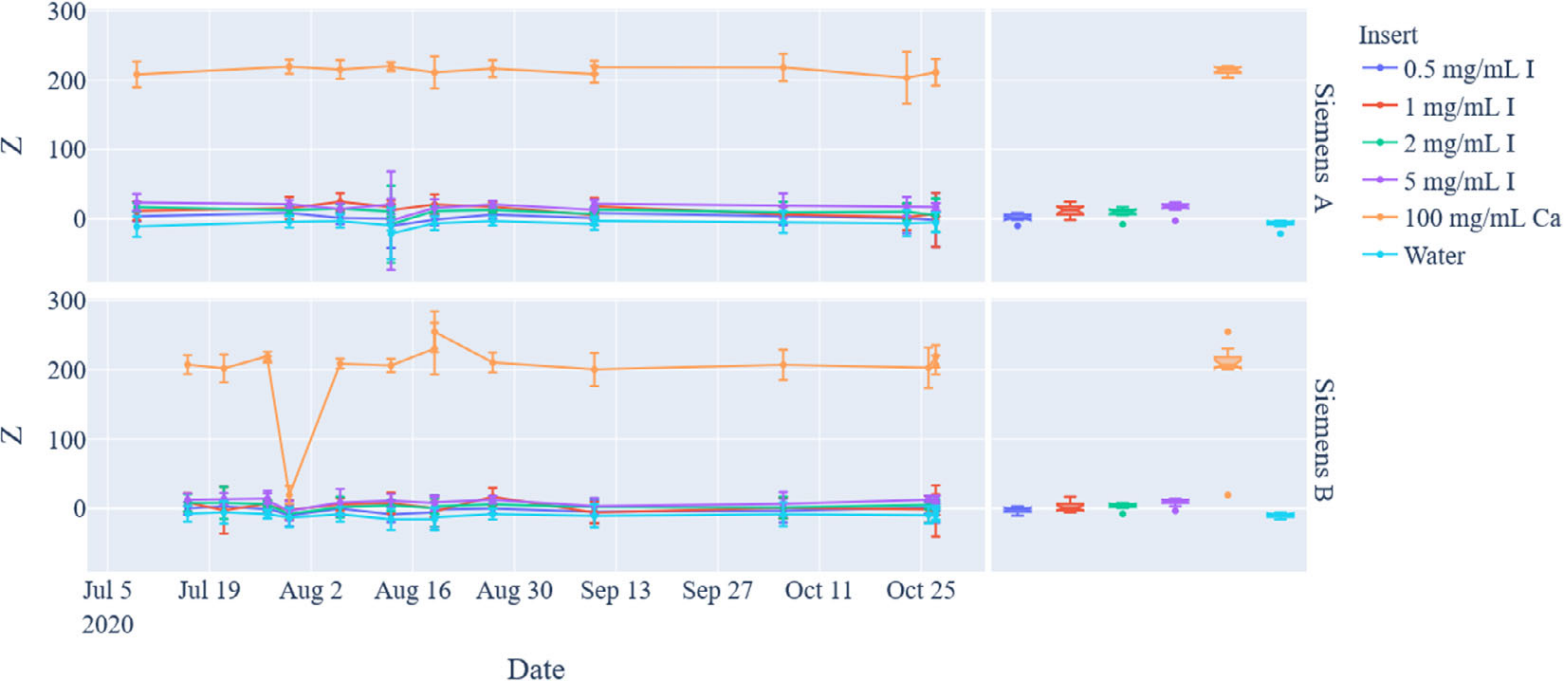
Dual‐energy quality control (DEQC) analysis of the dual‐energy computed tomography (DECT) phantom for virtual noncontrast (iodine‐subtracted images) (left) scatterplot showing mean mg/ml and standard deviation values for each insert over a 3‐month period (right) box plot to show overall measurement spread

Figure 7 and Table 5 show the DEQC results for VNC images. Based on the current automated workflow, this analysis can only be performed for the Siemens systems. The average HU values (mean ± SD) for VNC analysis measured across all systems over the 3‐month timeframe based on phantom insert were −9.6 ± 6.1 HU (0.5 mg I/ml), −2.3 ± 9.0 HU (1 mg I/ml), −6.1 ± 6.2 HU (2 mg I/ml), −5.7 ± 7.3 HU (5 mg I/ml), 220.1 ± 36.2 HU (100 mg Ca/ml), and −17.1 ± 4.4 (water equivalent material). The 33% decrease of the calcium measured HU in comparison to the mixed kV results is likely a result of calcium and iodine misclassification. Additionally, there are large deviations (up to 100%) with the iodine inserts. These deviations are not largely concerning since all the HU values from the iodine subtracted images are approximately less than 10 HU (near water background) in comparison to their larger HU values in the mixed kV and monoenergetic 70 keV images for these iodine inserts.

## 4 DISCUSSION

Our proof‐of‐concept study assessed the viability of a DECT phantom, protocol, and automated algorithm for routine DEQC. The DECT phantom introduced in this study was designed given constraints of cost and practicality. This study demonstrates a simple and affordable quality control program (phantom design, imaging protocol, and automated analysis software) to monitor clinical accuracy of DECT applications with a reduced scan field of view.

Future phantom designs will include the replacement of the 5 mg I/ml target insert with a mixed calcium/iodine rod at 2 mg I/ml and similar Ca concentration in terms of HU. This mixed rod would allow an additional level of quality assurance that is commonly seen in clinical practice to test and monitor the DECT system and corresponding reconstruction algorithms.

Of the trended data, there appears to be an extreme outlier (>3 interquartile range from the upper or lower limit of the statistical box plot) which occurred on 30 July for the Siemens Scanner B for the VNC image data on the 100 mg Ca/ml phantom insert. For the VNC outlier, the discrepancy was attributed to an error in the material decomposition algorithm misclassifying calcium for iodine; thus, subtracting calcium in the VNC image dataset. Likewise, the average measurement of the Ca phantom insert in the iodine map for that scanner on that day was 4.2 ± 0.4 mg I/ml, which is consistent with the other trending data values. This illustrates the importance of QC on all synthetic DECT image reconstructions, as satisfactory data from one image type (e.g., iodine quantification) does not necessarily mean satisfactory measurements in another image type (e.g., VNC). Additionally, to ensure clinical QA was unaffected, we checked VNC images in several patient studies from that scanner on the day the QC was taken as well as the day prior and after.

Furthermore, theoretically the iodine map should not be quantifying any amount of iodine for the calcium insert. However, as shown in Figure 6 and Table 5, a small portion of the calcium is being misclassified in the iodine quantification images due to imperfections in the material decomposition classifications. Similarly, we would expect in the VNC maps that the HU values of the Ca insert to remain above 300 HU (as shown in the mixed kV and monoenergetic images). However, as shown in Figure 7 and Table 5, the average HU value in the VNC images for the Ca insert is approximately 220 HU. The reduction in HU is another example of imperfect material decomposition. When implementing a DEQC program, it is important to note that these types of misclassifications are expected and are known limitations to DECT imaging. With better material decomposition algorithms or the use of additional (>2) CT energy sources (e.g., photon counting CT, multi‐energy CT), these effects may be diminished.

We recommend that the proposed phantom be scanned adjacent to the ACR or another phantom. This allows easy incorporation into a site's established routine ACR QC (i.e., biweekly and weekly) by simply adding an additional scan protocol for DEQC. Using an automated algorithm and QC databasing can allow the site to monitor other quality control aspects of DECT more routinely (i.e., image uniformity, low contrast detectability, spatial resolution). With our current protocol we found that when scanned alone, the results are susceptible to partial volume effects that may cause inaccurate measurements and ring artifacts as shown in Figure 2b. If the phantom is scanned alone, we recommend it be scanned in axial mode using a smaller beam collimation to reduce the likelihood of artifacts and inaccuracies. However, the use of a small beam width will limit the detection of possible issues from the peripheral detector row elements.

It is important to note the limitations based on the manufacturer system and DECT technology. For monoenergetic analysis, our study observed that GE tends to have less HU variation in comparison to the two Siemens systems. GE's technical manual states the tolerance for water is within 3 HU.^25^ While this threshold is accurate for the GE scanner, as shown, it does not hold true for the Siemens counterpart. Slightly larger variations can be observed with the two Siemens system consistent with Jacobsen's 12 HU bias over phantom inserts at the 70 keV monoenergetic energies.^8^ Similarly, Siemens reports a 10% ± 0.5 mg/ml error in iodine density measurements and also around a 10 HU differences between the VNC and true noncontrast images.^26^ For iodine density, Nute et al. found low error and low dependence for iodine density maps over 10 DECT scanners.^7^ Therefore, when implementing at other institutions there should be low variability in terms of iodine density based on differences in the DE protocol.

Additionally, a limitation to the proposed design is that the phantom does not contain an insert for uric acid, which would be beneficial for DECT applications for the diagnosis and characterization of gout^27^ and renal stones.^28^, ^29^ Like the proposed iodine/calcium mixed rod, a mixed insert of uric acid and calcium could be beneficial to ensure QC for gout and renal stone specific DECT applications. Another limitation to the current study is defining the action thresholds and recommendations for deviations in image measurements of the DECT phantom. As part of future works, we intend to deploy this DEQC methodology to more DECT capable scanners and monitor the results for an extended period (e.g., 6 months, 1 year). Thus, more trend analyses can show how DECT scanners are affected near times of system failures (i.e., X‐ray tube replacements) and recalibrations.

## 5 CONCLUSION

This work demonstrates a simple and affordable quality control program (phantom prototype, imaging protocol, and automated analysis software) to monitor clinical accuracy of DECT applications. The DEQC program has been implemented for three scanners over a 3‐month timeframe. Future works will include the addition of other DECT scanners that will be monitored over an extended timeframe (up to a year). With these additions, we expect to provide guidance and recommendations for DEQC action levels. With the addition of the described phantom, a site can easily incorporate DEQC use with current ACR QC procedures.

## CONFLICT OF INTEREST

A licensing agreement between Duke and Sun Nuclear is currently pending. Kenneth Ruchala is an employee of Sun Nuclear Corporation. Ehsan Samei has existing collaborations with GE and Siemens on studies with no bearing with the current paper.

## AUTHOR CONTRIBUTIONS

The above authors have contributed substantially to the concept, acquisition, analysis, and interpretation of this work. All authors have reviewed, approved, and agreed to be accountable for all aspects related to the accuracy integrity of this manuscript submission.
